# A General Synthetic Procedure for 2-chloromethyl-4(3*H*)-quinazolinone Derivatives and Their Utilization in the Preparation of Novel Anticancer Agents with 4-Anilinoquinazoline Scaffolds

**DOI:** 10.3390/molecules15129473

**Published:** 2010-12-22

**Authors:** Hong-Ze Li, Hai-Yun He, Yuan-Yuan Han, Xin Gu, Lin He, Qing-Rong Qi, Ying-Lan Zhao, Li Yang

**Affiliations:** 1 State Key Laboratory of Biotherapy and Cancer Center, West China Hospital, West China Medicinal School, Sichuan University, Chengdu 610041, China; 2 West China School of Pharmacy, Sichuan University, Chengdu 610041, China; 3 Department of Chemistry, Sichuan University, Chengdu 610064, China

**Keywords:** 2-chloromethyl-4(3*H*)-quinazolinones, anticancer activity, 4-anilino-quinazoline

## Abstract

In our ongoing research on novel anticancer agents with 4-anilinoquinazoline scaffolds, a series of novel 2-chloromethyl-4(3*H*)-quinazolinones were needed as key intermediates. An improved one-step synthesis of 2-chloromethyl-4(3*H*)-quinazolinones utilizing *o*-anthranilic acids as starting materials was described. Based on it, 2-hydroxy-methyl-4(3*H*)-quinazolinones were conveniently prepared in one pot. Moreover, two novel 4-anilinoquinazoline derivatives substituted with chloromethyl groups at the 2-position were synthesized and showed promising anticancer activity *in vitro*.

## 1. Introduction

**Figure 1:**
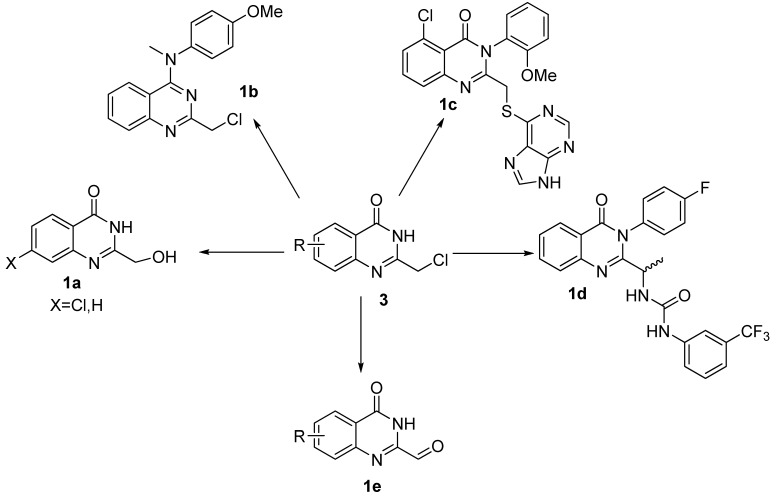
The versatile conversion of 2-chloromethyl-4(3*H*)-quinazolinones **3**.

2-Chloromethyl-4(3*H*)-quinazolinones are valuable intermediates in the preparations of a wide range of biologically active compounds such as anticancer agents **1a** [1] and **1b** [2], anti-inflammatory agent** 1c** [3], and hedgehog antagonist **1d** [4], *etc*. In addition, they also represent a class of functionalized and versatile building blocks, that is, these compounds can be converted into 2-hydroxymethyl-4(3*H*)-quinazolinones **1a**, 2-formyl-4(3*H*)-quinazolinones **1e**, *etc*. (Figure 1) 

Our research group has been interested in the design, screening, synthesis and biological evaluation of novel tumor growth inhibitors and apoptosis inducers as potential anticancer agents [5,6,7,8]. In our ongoing research of novel anticancer agents with 4-anilinoquinazoline scaffold, a series of novel 2-chloromethyl-4(3*H*)-quinazolinones **3 **are needed as key intermediates (Scheme 3).

**Figure 2:**
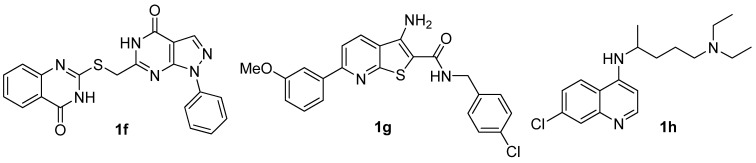
The representive hit compounds **1f**, **1g **and **1h** in our screening of anticancer agents.

**Scheme 1:**
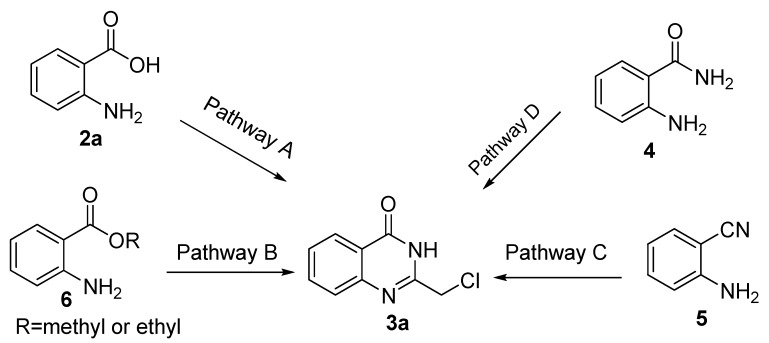
The reported routes toward **3a**.

Although a few methods for the synthesis of 2-chloromethyl-4(3*H*)-quinazolinones **3a** have been reported (Scheme 1), these methods (pathways A, B, C and D) all suffer from limitations. For example, only a few examples were reported without any systematic study of pathway A [9], the excessive amount usage of chloroacetonitrile (*ca.* 10 eq.) used under acid conditions in pathway B [10], the tedious route via oxidative annulation in pathway C [11] and the need for microwave irradition of pathway D [12]. Although pathway A has features that suggest high efficiency (only one step needed), mild reaction conditions, simple workup and purification, *etc.*, there has been no systematic study of this method. Thus, it is significant and attractive to develop a general procedure for the synthesis of 2-chloromethyl-4(3*H*)-quinazolinones **3. **

Herein, we wish to describe an improved synthetic procedure for 2-chloromethyl-4(3*H*)-quinazolinones **3** starting from *o*-anthranilic acids **2** (pathway A), and a convenient experimental protocol for the direct conversion of **3** to 2-hydroxymethyl-4(3*H*)-quinazolinones** i**n one flask, as well as their utilization in the preparations of novel anticancer agents.

## 2. Results and Discussion

### 2.1. Chemistry

Initial studies showed that many *o*-anthranilic acid substrates such as **2b** gave poor yields (up to 52%) via pathway A under literature conditions, although unsubstituted *o*-anthranilic acid **2a **gave a satisfactory yield (up to 88%). Therefore, **2b **was choosed as a model substrate for optimization of the reaction conditions and we set out to investigate the conditions for the condensation of **2b** with chloroacetonitrile (**11**) under different conditions. The results are shown in Table 1. The screening showed that the amount of chloroacetonitrile used was important for yield, and we were delighted to find that increasing the amount of chloroacetonitrile (from 1.0 to 3.0 equiv.) gave a much better yield (77%), as shown in entry 3. 

**Table 1:** The conditions optimization for substrate **2b**. 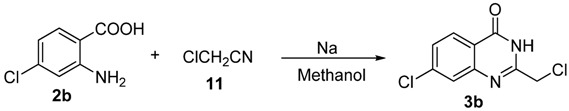

Entry	Temp (°C)	Substrates ratio ^a^ (2b:11)	Time (h)	Yield (%)
1	25	1:1	2	52
2	-	1:2	2	68
**3**	-	1:3	**2**	**77**
4	-	1:3	2	67^b^
5	-	1:4	4	78
6	50	1:3	2	76

Note: ^a ^All reaction run at 5 mmol scale; ^b ^The solvent is ethanol.

To demonstrate the generality of our procedure, we next investigated the condensation of a variety of *o*-anthranilic acids **2 **with chloroacetonitrile under the optimized conditions (1.0 equiv. anthranilic acid **2** and 3.0 equiv. chloroacetonitrile were reacted in methanol for 2 h at 25 °C). The results are presented in Table 2.

**Table 2:** The synthesis of 2-chloromethyl-4(3*H*)-quinazolinones **3**. 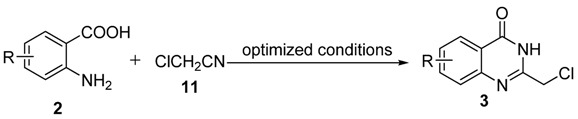

Entry	Substrate	Product	Yield (%)
1	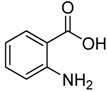 **2a**	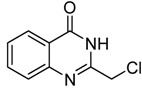 **3a**	88
2	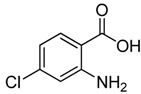 **2b**	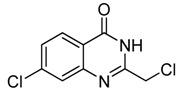 **3b**	76
3	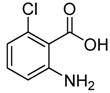 **2c**	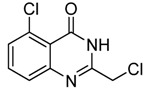 **3c^a^**	64
4	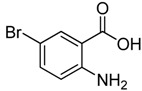 **2d**	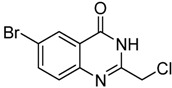 **3d**	84
5	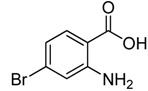 **2e**	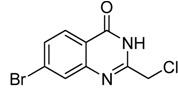 **3e**	75
6	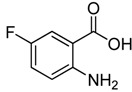 **2f**	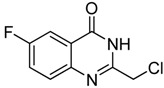 **3f**	77
7	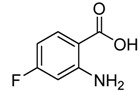 **2g**	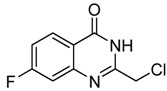 **3g**	72
8	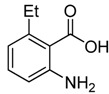 **2h**	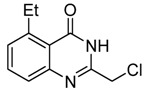 **3h^a^**	65
9	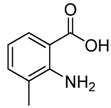 **2i**	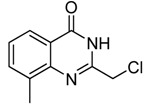 **3i**	58
10	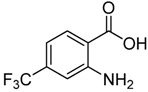 **2j**	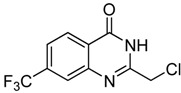 **3j**	70
11	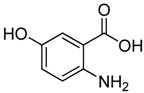 **2k**	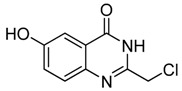 **3k^a^**	40
12	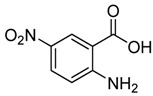 **2l**	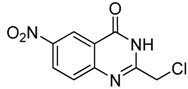 **3l^a^**	16
13	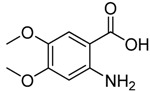 **2m**	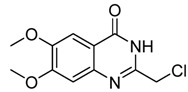 **3m**	78

**^a^** The product was a new compound.

As shown in Table 2, unsubstituted *o*-anthranilic acid **2a** (entry1) gave the best yield (88%). Reactions of substrates containing electron-withdrawing groups (entries 6-7, 10) and electron-donating groups (entry 13) on the phenyl ring gave comparable yields within the 70-78% range. Moreover, a slightly reduced yield was observed when the substitutent was adjacent to the amino and carboxylic groups (entries 3, 8-9). However, substrates with a hydroxyl and nitro group on the phenyl ring (entry 11, 12) gave only 40% and 16% yield, respectively, due to poor solubility in methanol. 

**Scheme 2:**
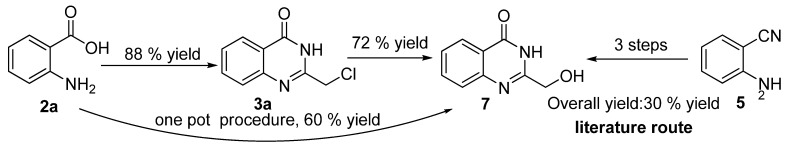
The one-pot synthetic route toward 2-hydroxymethyl-4(3*H*)-quinazolinone (**7**).

After the substrate extension, we next turned to synthesizing 2-hydroxymethyl-4(3*H*)-quinazolinone (**7**), a reported anticancer agent [1], with our procedure. To improve the operational efficiency, we speculated that the synthesis of **7** might be achieved in one flask without isolation and purification of **3a**. Gratifyingly, this one-flask procedure proceeded successfully and **7** was prepared from **2a** in two steps in one pot with 60% overall yield together with the virtue of simple purification (Scheme 2). It is noteworthy that the literature route needed three steps and gave 30% overall yield [1].

### 2.2. Anticancer activity

**Figure 3:**
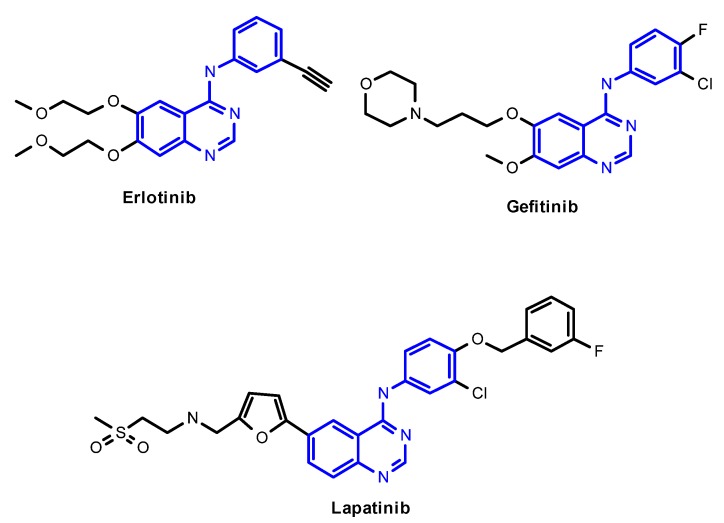
The structure of erolotinib, gefitinib and lapatinib.

**Scheme 3:**
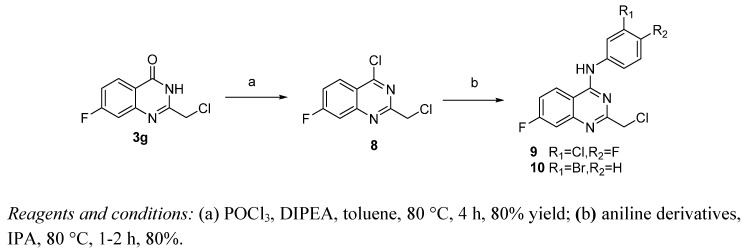
The synthetic route of **9** and **10**.

4-Anilinoquinazolines constitute an important class of protein kinase inhibitors approved by the US Food and Drug Administration (FDA) as marked drugs. Three of them with 4-anilinoquinazoline scaffolds (Figure 3), namely erolotinib (Tarceva®), gefitinib (Iressa®), lapatinib (Tykerb®) have achieved great clinical success [13]. The anticancer structure-activity relationships (SARs) of 4-anilinoquinazoline derivatives have been intensively studied, but the 2-position SAR has seldom been explored [2], so as part of our ongoing anticancer research on 4-anilinoquinazoline derivatives we focused on 2-position modification as shown in Scheme 3. 

Compounds **9 **and **10**, with 2-chloromethyl substituted 4-anilinoquinazoline scaffolds, were synthesized *via* chloration and condensation with aniline derivatives [2] starting from 2-chloromethyl-4(3*H*)-quinazolinone **3g **as shown in Scheme 3. 

The antiproliferative activities of compounds **9** and **10 **were examined in three different types of cancer cell lines including human hepatoma HepG2, breast cancer cell line MDA-MB-468, and colorectal cancer cell line HCT-116 using the MTT assay with gefitinib as the positive control. The experimental data are summarized in Table 3. 

**Table 3:** The anti-proliferative activities of compounds **9 **and **10** against various cancer cell lines.

Compound	IC_50 _(µM) ^a^		
	HepG2	MDA-MB-468	HCT-116
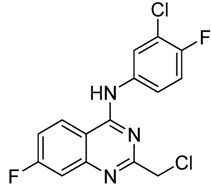 **9**	3.8	3.2	12.4
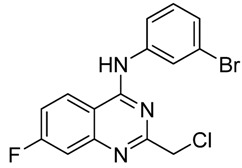 **10**	4.3	3.2	20
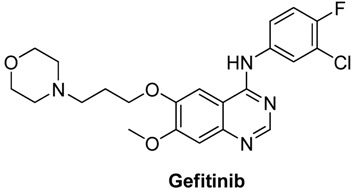	6.4	20	160

^a ^The cytotoxicity effects of compounds on various cancer cell lines were determined by the MTT assay. The results were expressed as the IC_50_, and were the means calculated from three independent experiments.

**Figure 4:**
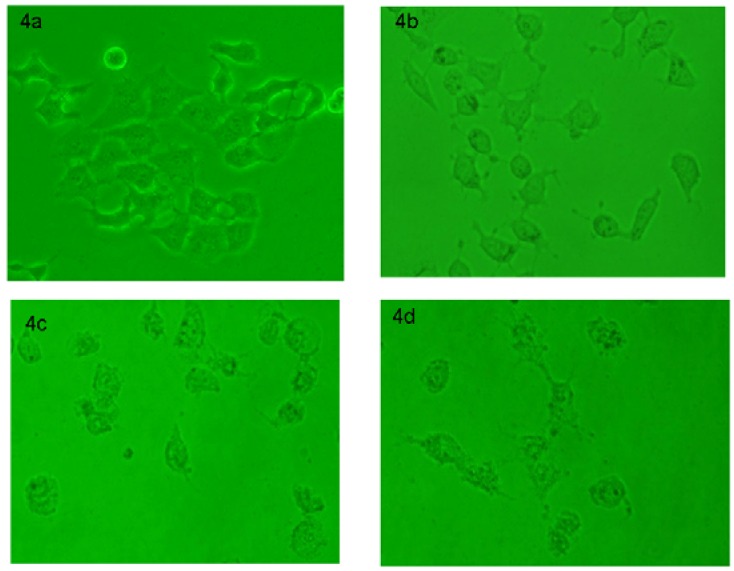
Morphology image of the HepG2 liver cancer cells treated with the control and compound **9 **(2.5-20 µM) for 24 h. (4a) the cells treated with DMSO 0.1% (v/v) as a vehicle control; (4b) 2.5 µM; (4c) 10 µM; and (4d) 20 µM.

Compounds **9 **and **10** show a broad-spectrum anti-cancer activity and low micromolar inhibition potency. It is noteworthy that the IC_50_ for HCT-116 cells was 12.4-20 µM, compared with an IC_50 _of 160 µM for gefitinib, the positive control. Besides the viability assay, the morphological changes were also studied under a contrast phase microscope as shown in Figure 4. Taking compound **9** for example, the obvious morphological changes of HepG2 cells were observed compared with the control (Figure 4a), after the treatment with compound **9** for 24 h (Figure 4b- Figure 4d). The results suggested that compound **9** might induce HepG2 cell death, because the cell shrinkage and rounding are common events in many cell death processes including both apoptosis and necrosis.

## 3. Experimental

### 3.1. General

All solvents and reagents were analytical grade pure and used without further purification. All melting points were determined on electric melting point apparatus and were uncorrected. ^1^H-NMR and ^13^C-NMR spectra were recorded on a Bruker Avance (Varian Unity Inova) 400 MHz spectrometer using TMS as internal reference chemical shift in δ, ppm. Low resolution ESI-MS spectra were carried out on a Waters triquadrupole mass spectrometer. High resolution Mass spectra were recorded on a Waters Q-TOF Premier mass spectrometer.

### 3.2. Typical procedure for the preparation of 2-chloromethylquinazolinones ***3a**-**3m***

To a flask containing sodium (23 mg, 1 mmol) was added anhydrous MeOH (5 mL), then chloroacetonitrile (0.95 mL, 15 mmol) with a syringe via a rubber septum, and the solution was stirred at ambient temperature for about 40 min under nitrogen. A solution of appropriate *o*-aminobenzoic acid **2 **(5 mmol) in anhydrous MeOH (25 mL) was then added. The reaction mixture was stirred at this temperature for about 2 h under nitrogen. The precipitate was collected by filtration, washed with MeOH (8 mL), H_2_O (8 mL) and MeOH (5 mL), respectively, and then dried under vacuum at room temperature to obtain the corresponding 2-chloromethylquinazolinone derivatives **3a-3l**. The following title compounds were prepared as described above:

*2-Chloromethylquinazolin-4(3H)-one* (**3a**). Prepared from *o*-anthranilic acid (**2a**) as a white solid; m.p. 249-250 °C; ^1^H-NMR (DMSO-d6): δ 12.59 (br s, 1H), 8.12 (dd, *J* = 1.2, 7.2 Hz, 1H), 7.84 (dd, *J* = 1.2, 7.2 Hz, 1H), 7.68 (d, *J* = 8.0 Hz, 1H), 7.56 (t, *J* = 8.0 Hz, 1H), 4.55 (s, 2H); ^13^C-NMR (DMSO*-*d6): δ 161.79, 152.62, 148.47, 134.88, 127.52, 126.14, 121.52, 106.89, 43.53; ESI-MS: m/z 195.03 (M+H^+^).

*2-Chloromethyl-7-chloroquinazolin-4(3H)-one* (**3b**). Prepared from 7-chloro-2-aminobenzoic acid (**2b**) as a white solid; m.p. 232-234 °C; ^1^H-NMR (DMSO-d6): δ 12.72 (br s, 1H), 8.10 (d, *J* = 8 Hz, 1H), 7.75 (s, 1H), 7.58 (d, *J* = 8 Hz, 1H), 4.55 (s, 2H); ^13^C-NMR (DMSO*-*d6): δ 161.15, 154.13, 149.53, 139.44, 128.10, 127.68, 126.64, 120.30, 43.29; ESI-MS: m/z 227.05 (M-H^+^).

*2-Chloromethyl-5-chloroquinazolin-4(3H)-one* (**3c**). Prepared from 6-chloro-2-aminobenzoic acid (**2c**) as a white solid; m.p. 220-221 °C; ^1^H-NMR (DMSO-*d*_6_): δ 12.63 (br s, 1H), 7.74 (t, *J* = 8.0 Hz, 1H), 7.60 (d, *J* = 8.0 Hz, 1H), 7.54 (d, *J* = 8.0 Hz, 1H), 4.51 (s, 2H); ^13^C-NMR (DMSO*-*d6): δ 159.87, 153.35, 150.91, 134.56, 132.78, 129.76, 127.00, 118.42, 42.97; ESI-MS: m/z 227.08 (M-H^+^).

*2-Chloromethyl-6-bromoquinazolin-4(3H)-one* (**3d**). Prepared from 5-bromo-2-aminobenzoic acid (**2d**) as a white solid; m.p. 240-242 °C; ^1^H-NMR (DMSO-d_6_): δ 12.80 (br s, 1H), 8.19 (d, *J* = 2.0 Hz, 1H), 7.98 (dd, *J* = 2.4, 8.0 Hz, 1H), 7.63 (d, *J* = 8.0 Hz, 1H), 4.55 (s, 2H); ^13^C-NMR (DMSO*-*d6): δ 160.62, 153.21, 147.37, 137.63, 129.80, 128.23, 123.07, 119.97, 43.34; ESI-MS: m/z 271.01 (M-H^+^).

*2-Chloromethyl-7-bromoquinazolin-4(3H)-one* (**3e**). Prepared from 4-bromo-2-aminobenzoic acid (**2e**) as a white solid; m.p. 238-240 °C; ^1^H-NMR (DMSO-d6): δ 12.77 (br s, 1H), 8.02 (d, *J* = 8.0 Hz, 1H), 7.91 (s, 1H), 7.72 (d, *J* = 8.0 Hz, 1H), 4.55 (s, 2H); ^13^C-NMR (DMSO*-*d6): δ 161.24, 154.01, 149.56, 130.42, 129.67, 128.38, 128.10, 120.55, 43.28; ESI-MS: m/z 270.99 (M-H^+^).

*2-Chloromethyl-6-fluoroquinazolin-4(3H)-one* (**3f**). Prepared from 5-fluoro-2-aminobenzoic acid (**2f**) as a white solid; m.p. 239-241 °C; ^1^H-NMR (DMSO-d6): δ 12.75 (br s, 1H), 7.76 (m,* J* = 8.0 Hz, 3H), 4.56 (s, 2H); ^13^C-NMR (DMSO*-*d6): δ 161.81, 161.17, 159.36, 152.06, 145.27, 130.35, 123.36, 123.00, 122.80, 110.91, 43.40; ESI-MS: m/z 211.06 (M-H^+^).

*2-Chloromethyl-7-fluoroquinazolin-4(3H)-one* (**3g**). Prepared from 7-fluoro-2-aminobenzoic acid (**2g**) as a white solid; m.p. 247-249 °C; ^1^H-NMR (DMSO-d6): δ 12.72 (br s, 1H), 8.18 (dd, *J* = 2.4, 8.0 Hz, 1H), 7.48 (dd,* J* = 2.0, 8.0 Hz, 1H), 7.42 (dd, *J* = 2.4, 8.0 Hz, 1H), 4.55 (s, 2H); ^13^C-NMR (DMSO*-*d6): δ 167.19, 164.70, 161.04, 154.07, 150.58, 129.15, 118.45, 115.83, 112.57, 43.25; ESI-MS: m/z 213.03 (M+H^+^).

*2-Chloromethyl-5-ethylquinazolin-4(3H)-one* (**3h**). Prepared from 6-ethyl-2-aminobenzoic acid (**2h**) as a white solid; m.p. 221-222 °C; ^1^H-NMR (DMSO-d6): δ 12.34 (br s, 1H), 7.69 (t, *J* = 8.0 Hz, 1H), 7.49 (d, *J* = 8.0 Hz, 1H), 7.30 (d, *J* = 8.0 Hz, 1H), 4.52 (s, 2H), 3.24 (q, *J* = 7.2 Hz, 2H), 1.19 (t, *J* = 7.2 Hz, 3H); ^13^C-NMR (DMSO*-*d6): δ 162.01, 152.11, 150.19, 140.63, 134.09, 128.54, 125.80, 119.03, 43.14，27.87, 16.46; ESI-MS: m/z 223.11 (M+H^+^).

*2-Chloromethyl-8-methylquinazolin-4(3H)-one* (**3i**). Prepared from 3-methyl-2-aminobenzoic acid (**2i**) as a white solid; m.p. 246-248 °C; ^1^H-NMR (DMSO-d6): δ 12.59 (br s, 1H), 7.96 (d, *J* = 7.6 Hz, 1H), 7.69 (d, *J* = 7.2 Hz, 1H), 7.41 (t, *J* = 7.6 Hz, 1H), 4.56 (s, 2H); ^13^C-NMR (DMSO*-*d6): 162.05, 151.40, 146.88, 135.76, 135.28, 126.95, 123.75, 121.46, 43.83, 17.39; ESI-MS: m/z 207.05 (M-H^+^).

*2-Chloromethyl-7-trifluoromethylquinazolin-4(3H)-one* (**3j**). Prepared from 4-trifluoromethyl-2-aminobenzoic acid (**2j**) as a white solid; m.p. 195-197 °C; ^1^H-NMR (DMSO-d6): δ 12.90 (br s, 1H), 8.31 (d, *J* = 8.0 Hz, 1H), 8.01 (s, 1H), 7.84 (d, *J* = 8.0 Hz, 1H), 4.58 (s, 2H); ^13^C-NMR (DMSO-d6): δ 168.95, 161.04, 154.38, 151.61, 148.50, 134.30, 127.81, 124.61, 124.34, 71.62, 43.24; ESI-MS: m/z 261.01 (M-H^+^).

*2-Chloromethyl-6-hydroxyquinazolin-4(*3*H)-one* (**3k**). Prepared from 5-hydroxy-2-aminobenzoic acid (**2k**) as a white solid; m.p. > 250 °C; ^1^H-NMR (DMSO-d6): δ 12.40 (br s, 1H), 10.52 (s, 1H), 7.54 (d, *J* = 8.4 Hz, 1H), 7.40 (d, *J* = 2.4 Hz, 1H), 7.26 (dd, *J* = 2.4, 8.4 Hz, 1H), 4.51 (s, 2H); ^13^C-NMR (DMSO*-*d6): δ 161.59, 156.29, 149.20, 141.52, 129.21, 124.25, 122.51, 109.17, 43.70; ESI-MS: m/z 209.02 (M-H^+^).

*2-Chloromethyl-6-nitroquinazolin-4(3H)-one* (**3l**). Prepared from 5-nitro-2-aminobenzoic acid (**2l**) as a white solid; m.p. 240-241 °C; ^1^H-NMR (DMSO-d6): δ 13.10 (br s, 1H), 8.79 (d, *J* = 2.0 Hz, 1H), 8.55 (dd, *J* = 2.4, 8.0 Hz, 1H), 7.87 (d, *J* = 8.0 Hz, 1H), 4.60 (s, 2H); ^13^C NMR (DMSO*-*d6): δ 161.07, 156.31, 152.64, 145.50, 129.33, 122.31, 121.67, 43.25; ESI-MS: m/z 240.11 (M+H^+^).

*2-Chloromethyl-6,7-dimethoxyquinazolin-4(3H)-one* (**3m**). Prepared from 2-amino-4,5-dimethoxy-benzoic acid (**2m**) as a white solid; m.p. 240-241 °C; ^1^H-NMR (DMSO-d6): δ 12.30 (br s, 1H), 8.68 (s, 1H), 8.34 (s, 1H), 4.54 (s, 2H), 3.97 (s, 3H), 3.83(s, 3H); ^13^C-NMR (DMSO*-*d6): δ 162.04, 157.31, 153.65, 146.54, 128.73, 120.37, 118.87, 56.12, 54.25, 43.15; ESI-MS: m/z 255.01 (M+H^+^).

### 3.3. One-pot synthesis of 2-hydroxymethylquiazolin-4(3H)-one *(**7**)*

To a flask containing sodium (23 mg, 1 mmol) was added anhydrous MeOH (5 mL), then chloroacetonitrile (0.95 mL, 15 mmol) with a syringe via a rubber septum, and the solution was stirred at ambient temperature for about 40 min under nitrogen. A solution of o-aminobenzoic acid (686 mg, 5 mmol) in anhydrous MeOH (25 mL) was then added. The reaction mixture was stirred at this temperature for about 2 h under nitrogen, the execess MeOH was removed by evaporation in vaccum, and the residue was added aqueous NaOH solution (2 mol/L, 10 mL), the mixture was stirred under reflux for 1 h. After cooling to room temperature, the reaction mixture was neutralized with dil. HCl solution, the precipitates were filtered, washed with cold MeOH to give the title compound **7** as a white solid, yield: 60%; m.p. 278-279 °C; ^1^H-NMR (DMSO-d6): δ 11.94 (br s, 1H), 8.11 (d, *J* = 8 Hz, 1H), 7.82 (t , *J* = 7.2 Hz, 1H), 7.64 (d, *J* = 8 Hz, 1H), 7.51 (d, *J* = 7.2 Hz, 1H), 5.60 (s, 1H), 4.39 (s, 1H); ^13^C-NMR (DMSO*-*d6): δ 161.75, 157.05, 148.84, 143.60, 134.67, 127.16, 126.14, 121.54, 61.86; ESI-MS: m/z 176.92 (M+H^+^).

### 3.4. General procedure for the preparation of 4-anilinoquinazoline derivatives ***9*** and ***10***

A mixture of 2-chloromethyl-7-fluoroquinazolin-4(3*H*)-one (**3g**, 2.551 g, 12 mmol) in anhydrous toluene (60 mL) and DIPEA (4.2 mL, 24 mmol) contained in a 100 mL flask equipped with a condenser and a drying tube was refluxed for 40 min. After cooling to room temperature and the addition of POCl_3_ (2.2 mL, 24 mmol), the mixture was heated at 80 °C for 4 h. After cooling to room temperature, the mixture was diluted with ethyl acetate (100 mL), washed successively with water, saturated NaHCO_3_ and brine, the organic layer was dried by MgSO_4_, filtered and concentrated to obtain the intermediate **8**. The mixture of intermediate **8** (480 mg, 2 mmol) and appropriate aniline (2.4 mmol) in isopropanol (20 mL) was stirred at 60 °C for 2-3 h and cooled to room temperature, the precipitates were collected by filtration, washed with cold isopropanol, and dried at room temperature for 24 h under vacuum to provide the corresponding product **9** or **10**.

*N-(3-chloro-4-fluorophenyl)-2-(chloromethyl)-7-fluoro-3,4-dihydroquinazolin-4-amine hydrochloride* (**9**). Yellow solid, yield: 73%; m.p. 204-206 °C; ^1^H-NMR (DMSO-d6): δ 11.31 (br s, 1H), 9.02 (t, *J* = 7.2 Hz, 1H), 8.28 (dd,* J* = 2.4, 7.2 Hz, 1H), 7.93 (m, 1H), 7.74 (m, 2H), 7.52 (t, *J* = 8.8 Hz, 1H), 4.85 (s, 2H); ^13^C-NMR (DMSO*-*d6): δ 167.07, 164.53, 160.26, 158.88, 156.20, 153.76, 134.56, 128.88, 125.81, 124.48, 119.37, 117.87, 110.32, 107.00, 43.81; ESI-MS: m/z 340.07 (M+H^+^).

*N-(3-bromophenyl)-2-(chloromethyl)-7-fluoro-3,4-dihydroquinazolin-4-amine hydrochloride* (**10**). Straw yellow solid, yield: 75%; m.p. 208-210 °C; ^1^H-NMR (DMSO-d6): δ 10.88 (br, s, 1H), 8.92 (t,* J* = 7.6 Hz, 1H), 8.32 (s, 1H), 7.96 (m, 1H), 7.74 (dd, *J* = 2.4, 7.6 Hz, 2H), 7.42 (d,* J* = 7.6 Hz, 2H), 4.82 (s, 2H); ^13^C-NMR (DMSO*-*d6): δ 167.20, 164.66, 160.33, 159.07, 139.05, 130.78, 129.06, 128.81, 126.60, 122.87, 121.50, 118.02, 110.51, 107.06, 43.86; ESI-MS: m/z 366.03 (M+H^+^).

### 3.5. In vitro assay for cytotoxic activity (MTT assay)

Materials

Methylthiazolyldiphenyl-tetrazolium bromide (MTT), dimethyl sulfoxide (DMSO) were purchased from Sigma (St. Louis, MO, USA). HepG2 (ATCC Accession No. HB-8065), MDA-MB-468 (ATCC Accession No. HTB-132) and HCT-116 (ATCC Accession No. CCL-247) were obtained from American Type Culture Collection (ATCC, Rockville, MD, USA) and were grown as monolayers in Dulbecco’s modified Eagle medium or RPMI 1640 medium (Gibco BRL, Grand Island, N.Y., USA). These cells were supplemented with 10% heat-inactivated fetal bovine serum (FBS; Gibco, Auckland, N.Z.), 100 units/mL penicillin, 100 units/mL streptomycin, at 37 °C, 95% relative humidity, under 5% CO_2_. The cytotoxicity effects of these compounds on various cancer cells were determined by the MTT assay, and the results were expressed as the IC_50_, that were means calculated from three independent experiments. Briefly, cells (2000/well) were seeded in 96-well plates and cultured for 24 h, followed by treatment with the compounds for 48 h. Ten microliters of 10 mg/mL MTT was added per well and incubated for another 2.5 h at 37 °C. Then the supernatant fluid was removed and 150 μL/well DMSO was added for 15-20 minutes. The absorbance (OD) of each well was measured at 570 nm, using a SpectraMAX M5 microplate spectrophotometer (Molecular Devices).

## 4. Conclusions

In conclusion, an improved and efficient procedure for the synthesis of 2-chloromethyl-4(3*H*)-quinazolinones **3** has been described. The results indicated that: 1) increasing the amount of chloroacetonitrile improved the yields remarkably; 2) the convenient one-pot process toward 2-hydroxymethyl-4(3*H*)-quinazolinone allowed the two-step reaction could be done in a single reaction vessel, without isolation and purification the intermediate other than a simple filtration and washing. Moreover, two 4-anilinoquinazoline analogs substituted with chloromethyl groups at the 2-position were synthesized and showed promising anticancer activity *in vitro*, which deserves further studies.
